# Etiology of Pyorrhœa Alveolaris

**Published:** 1894-01

**Authors:** C. N. Peirce


### International Dental Journal.

Vol. XV. January, 1894. No. 1.

### Original Communications.[1]

### ETIOLOGY OF PYORRHŒA ALVEOLARIS.[2]

BY C. N. PEIRCE, D.D.S.

Mr. Chairman and Members of the New York Odontological Society,—I have no apology to offer for my presence here this evening. I come, if I understand it correctly, in response to your invitation, seconded by my interest in the subject under consideration. Your committee must, therefore, assume some of the responsibility regarding the time I shall occupy, that is, whether it shall be profitable or otherwise. The title of the short paper I shall present to you is that of the "Etiology of Pyorrhœa Alveolaris." It can certainly be truthfully stated that the profession, or such members of it who have written upon the pathology of this disease, are far from being united in their views.

In April, 1892, there was published in the International Dental Journal an essay on this subject. If there was in that communication truth or falsehood, I shall not disown it, but shall endeavor to-night to bring further evidence to substantiate the more important statements therein contained. It appears to me that

[1] The editor and publishers are not responsible for the views of authors of papers published in this department, nor for any claim to novelty, or otherwise, that may be made by them. No papers will be received for this department that have appeared in any other journal published in the country.

[2] Read before the New York Odontological Society, November 21, 1893. The publication of this paper is in advance of the discussion, which will appear in the next issue.

there is always an advantage to be gained when great obscurity of thought prevails as to the true nature of any complex morbid state, such as we witness in pyorrhœa alveolaris, to endeavor as far as possible, to reduce it to its simplest factors, and, next, to determine the primary origin of each, whether local or constitutional.

The pathological state affords the only true basis for a scientific classification of diseases. The term pyorrhœa alveolaris refers only to one symptom, and is, therefore, provisional, and more or less objectionable. As you are all aware, Dr. G. V. Black has, with some degree of appropriateness, applied to the disease under consideration the terms gingivitis or phagedenic pericementitis and calcic pericementitis. While these two terms approximate the truth, they do not, to my mind, express the whole truth. From a careful study of the abnormal pericemental or rather the alveolo-cemental membrane, it appears to me that we must recognize two closely-allied, but yet very different pathological states; different, as I shall attempt to demonstrate, in their *etiology*, in their *clinical history*, in their *symptomatology*, and in their *susceptibility to treatment.* In order the more clearly to convey my conception as to the causation, the evolution, and the differentiation of the varieties of calcic pericementitis, it has been found necessary to coin two terms, which, it is believed, will be more expressive as to their true nature, and for which you will pardon me I trust. In the first place, I believe that while pericementitis is associated with calcic deposit, the origin of the calcic salt and the antecedent condition which determines the locality and character of the deposit, as well as the train of totally distinct symptoms which follow, lead inevitably to the conclusion, that two different diseases have thus far been confounded. In one form of pericementitis the origin of the calcic salt is the saliva, and in the other form, the blood. The former I shall designate as *ptyalogenic* calcic pericementitis, expressive of the idea that in its origin it is *local*, *peripheral*, and *salivary.* The latter I shall designate as *hœmatogenic* calcic pericementitis, expressive of the idea that in its origin it is *constitutional*, *central*, and *associated* with some modification of the normal composite of the blood plasma.

I trust that when the pathological specimens with which you are familiar, and a few of which I have with me, are carefully compared, the product analyzed, and the clinical histories and symptoms of these two diseases contrasted, the appropriateness of this division and the terms employed to designate them will become apparent and reconcilable.

That hæmotogenic calcic pericementitis is an altogether distinct affection from the preceding, and dependent for its cause upon some morbid material derived from the blood, will, I think, become apparent from the facts which I hope to adduce.

Let it be understood that by this term reference is made to the disease with which we are all familiar as *pyorrhœa alveolaris.* The symptoms of this affection are too familiar to us all to detain us more than a few moments. Let it be recalled that in this form of the disease the morbid process begins on the root, and very frequently, if not usually, in the vicinity of the apical extremity, this being in marked contrast to the ptyalogenic form, which always has its origin at the gingival borders; that the inflammatory process is attended with pain, swelling, tissue disintegration, and the formation of pus; that there is a deposition of some insoluble salt upon the cementum, which, as it accumulates, eventually detaches the cemental membrane; that the alveolar process becomes absorbed; that the root of the tooth becomes horny in appearance, etc. These symptoms, taken in connection with the fact that thus far in the curative effort the disease has resisted all forms of local treatment, whether manipulative or therapeutic, it seems, would lead us inevitably to the conclusion that we have here a distinct disease of constitutional origin.

In ptyalogenic pericementitis it has long been recognized that the exciting cause is the deposition of calcium phosphate and carbonate around the gingival borders, which deposition infiltrating itself into the alveolo-cemental membrane is the immediate cause of the subsequent inflammation and its concomitants. Without any apparent reason it has always been assumed that in the hæmatogenic form, the immediate cause was also the deposition of similar calcic salts. The fact, however, that in true pyorrhœa the symptoms are so different, and that all local treatment has been practically so unavailing, it has suggested the possibility that some other chemical agent derived from the blood, and the product of some morbid constitutional state, might be the exciting cause.

With the view of testing the plausibility of this assumption, I have had the deposit removed from the apical extremities of teeth which have been sacrificed by this so-called pyorrhœa, and subjected to chemical analysis. In every instance the chemical methods employed revealed the fact that the deposit was a combination of calcic urate, sodic urate, with some calcic phosphate and carbonate. The existence of the urates in which the uric acid is the predominating element shows that this deposit is a precipitate from blood exudation, and the irritation of constitutional origin, and the disease pyorrhœa alveolaris but another phase of the uric-acid or gouty diathesis. It was discovered some years ago by Dr. Garrod, an English physician, that the blood plasma of gouty patients contains an excess of uric acid amounting to 0.11 grain ($\frac{1}{10}$ of a grain) in 1000 grains of blood plasma. Other excrementitious substances closely allied to uric acid are also present in the blood. Whatever view may be taken as to the origin of the uric acid, whether it is due to an imperfect oxidation of the food constituents, or whether it is the product of abnormal *metabolism* of protoplasmic tissue, or to faulty innervation, the fact remains, that in this diathesis there is an abnormal amount of this acid and its salts in the blood. Owing to the non-diffusibility of uric acid, it is but imperfectly eliminated from the blood by the kidneys. Accumulating in consequence, it enters into combination with sodium and calcium, forming urates. When the amount of uric-acid salts attains a certain percentage they are eliminated from the blood through the walls of the capillary blood-vessels, passing out associated with the lymph. The tissues which become the seat of this salt-exudation are pre-eminently the connective tissues; those tissues presenting the greatest degree of density and the least degree of vascularity. Hence the tissues forming joints become the primary seats of the uric acid deposits. It must, however, be remembered that all tissues, or rather the same tissues in all parts of the body may be thus affected.

The salts found in the exudation of gouty subjects are usually urates of lime, soda, and, to some extent, the phosphate and carbonate of lime. After the fluid has been exuded from the vessels, the non-appropriated lymph is absorbed by the lymphatics, and the salts left behind are deposited in both the amorphous and crystalline forms. The presence of the salts, acting as foreign bodies, excite the ordinary phenomena of inflammation.

Let us now apply these facts to the disease in question,—viz., pyorrhœa alveolaris. Inasmuch as all portions of the body have been shown by pathologists to be liable to uric acid deposits, it is not at all strange that the alveolo-cemental membrane, composed largely of connective tissue, should also become a depot for uric-acid deposits. It is more than probable that as a predisposing cause there might co-exist some impairment in the nutrition of this membrane dependent upon either local mechanical force, or some obscure faulty innervation.[1] However this may be, the mere presence of these salts leads to the conclusion that here as elsewhere they

---

[1] Wedging, malleting, etc.

are derived from the blood by or through the medium of the lymph stream. With the absorption of the excess of lymph, the residual salts become precipitated upon the cemental surface.

It is for this reason that I regard the deposition of uric acid as of blood origin and the disease pyorrhœa alveolaris as one of the local manifestations of the constitutional state familiar to all pathologists as the uric-acid or gouty diathesis. Assuming now that the deposition of uric-acid salts is the primary cause of this form of pericementitis, what would naturally be the successive stages in its evolution?

As the current of the lymph-stream is directed for the most part towards the cementum, through its borders or periphery into the lacunæ and canaliculi, and finally in the reverse direction, it is not difficult to see why the deposit should take place on the surface of the cementum as well as in the meshes of the alveolo-cemental membrane. The constant deposition and pressure of these insoluble salts will act as irritants engendering the well-known inflammatory states,—viz., congestion, exudation, impaired nutrition, tissue disorganization, and the formation of pus. These changes taking place here as elsewhere in the immediate vicinity of the irritation —that is, on the cemental aspect of the membrane—leads to its detachment from the cementum and the development of a pus-pocket.

As the disease is progressive, extending through a series of years, it might be expected that the deposition of these uric-acid salts would attain a greater volume than is generally found on most affected teeth; but it must be remembered that, although these salts are insoluble, as long as fluid in the form of pus is present, their crystallization and constant accumulative deposition is largely prevented by being carried away by the pus-stream which flows through the pocket or sinus to the gum margins. An examination of this pus has not yet been effected, but there is little doubt, when it is, that it will demonstrate the presence of the urates. Now, how will this interpretation of the pathology of pyorrhœa alveolaris account for that other symptom which is so characteristic a feature,—viz., disappearance of the alveolar process. The key to the mechanism of alveolar absorption is furnished by the pathology of bone-softening in general. If the periosteum of any bone becomes the seat of inflammation, an exudation is poured out from its inner surface, which is in close contact with the compact tissue. This exudation exerts pressure on the bone, interferes with its nutrition, and, in consequence, leads to softening and absorption. If the tension be not relieved by the removal of the exudation, the softening increases and necrosis results, but if the pressure be removed in sufficient time, the progressive pathological state never passes beyond the stage of softening and absorption.

In pericementitis the effusion exerts a pressure in both directions,—towards the cementum and towards the alveolar process. The constant pressure of the exudation would, by interference with the nutrition of the process, lead not only to softening and absorption, but to necrosis also, if it were not that as the pus accumulates and the pressure rises the fluid takes the line of the least resistance and burrows towards the gum margins, and so relieves the pressure on the alveolar process before complete strangulation of the tissues takes place; but as the pressure from pus accumulation rises and falls, from time to time, there will be periodical compressions with some pain and gradual absorption. If necrosis of the process should, however, occur in any appreciable amount, we should have it demonstrated by exfoliation and sequestration.

The pus, to which allusion has already been made, is doubtless effected through the influence of the micro-organisms upon the exudation. In the multiplication of these bacteria they increase the disintegration of tissues and so contribute to the establishment of this pus-containing cavity or pocket,—an abscess, from which, without treatment, pus is continually discharged until the surrounding tissues are destroyed and the tooth either drops out or, to relieve discomfort, is artificially removed. The staphylococcus, which is supposed to be the bacterial form present, is not considered pathogenic, though experiments made by Dr. G. V. Black led him to believe that it was not a passive or harmless concomitant of this purulent inflammatory process.

I have had three specimens of this tooth-deposit examined chemically by Professor Ernest Congdon, of the Drexel Institute, whose experimental skill is a sufficient guarantee for the accuracy of the results obtained.

The tests which were employed were: 1, the hydrochloric acid test; 2, the dry or the destructive distillation test; 3, the murexide test. The value of the hydrochloric-acid test depends upon the fact that the acid has the power of breaking up the compounds of uric acid and separating crystals of uric acid.

After the removal from the root of the tooth the deposit was placed in a watch-glass and mixed with a few drops of distilled water; to this several drops of commercially pure hydrochloric acid were added and the glass placed over the sand-bath of a micro-chemical burner and subjected to a moderate heat for some time, or until the solution had evaporated to dryness, when the residue was examined microscopically.

The dry or destructive distillation test depends for its value upon the development of ammonia. The molecular composition of uric acid is $C_5H_4N_4O_3$. When this acid or any of its salts is decomposed by heat, the atoms are disassociated and ammonia gas, $NH_3$, a strongly alkaline compound, is set free. A few grains of this deposit being placed in the end of a test-tube and a highly sensitive red litmus-paper moistened with distilled water and placed in the open end and then subjected to heat, the resulting ammonia gas ascends and in a few minutes colors the litmus-paper deep blue. As no other alkali is volatile, this is regarded as an extremely delicate test for ammonia, and as this can only come from a nitrogen-holding compound,—as urea, uric acid, or an allied compound,—the conclusion is inevitable that we have had present in the test-tube one of the just-mentioned compounds.

The murexide test is the usual one for uric acid and its salts. The deposit, being placed in a watch-glass or porcelain cup, is treated to a few drops of strong nitric acid; decomposition at once takes place, and a yellow color is produced. Nitrogen and carbon dioxide gases are given off; urea and alloxan remain behind. The solution being slowly evaporated, the residue is allowed to cool; upon the addition of a few drops of dilute ammonia, if uric acid is present, a purplish-red color—"*murexide*"—is at once produced. Three specimens were examined by Professor Congdon by these three tests, and the following results were reported to me by him:

Specimen No. 1 contained, as shown by microscopical analysis, a number of fine needle-crystals of calcium urate, a few crystals of free uric acid, and crystals of calcium phosphate. Destructive distillation analysis yielded a strong ammonia reaction. The murexide test for uric acid and its compounds was faint, though the characteristic color showed in several places.

Specimen No. 2 presented the same crystals on microscopical investigation. The murexide test was strong, producing a number of purplish-red spots.

Specimen No. 3 yielded similar results.

In addition to these three analyses by Professor Congdon, some six or eight specimens were examined by my friend and colleague Professor A. P. Brubaker, M.D., D.D.S., in my library and in my presence, the results obtained corresponding to those of Professor

Congdon. In three of these an abundance of urate of soda crystals were observed. It must be remembered that, as the quantity embraced in each specimen was small in amount, large results could scarcely be obtained or expected.

The treatment of the constitutional state, of which the pericementitis is but a local expression, resolves itself into,—1, the prevention of the uric-acid formation; 2, the relief of the lesions occasioned by its depositions. Uric acid, we are led to believe, is one of the many products which result from the destructive and imperfect metabolism of nitrogenized food. When all foods are completely oxidized in the body, the ultimate compounds are carbon dioxide, urea, and water. Large consumption of foods, over-indulgence in alcoholic and malt liquors, insufficient exercise, form a combination of causes which prevent complete oxidation of foods and encourage the development of numerous side products, among which are uric acid and its salts. Among foods it has been thought that the nitrogenized were the chief sources of uric acid, and, while this is undoubtedly the case, it is also a well-known fact that the saccharine and starchy foods, by occasioning gastric and intestinal derangement, are in all probability the remote causes of the subsequent imperfect digestion of the albuminous foods. As far as dietetic treatment is concerned, the regular practitioner feels that in treating uric-acid disorders attention must be paid to the existing digestive condition, to the regulation of diet, to the oxidation and elimination of waste products, by out-door exercise, by the drinking of hot water, by the use of alkaline and saline waters, such as Apollinaris, Hunyadi, Marienbad, Buffalo Lithia, etc. The administration of alkalies and iron, agents which improve the digestion and promote oxidation, have also proved of benefit. It must be remembered, however, that this constitutional vice is not established suddenly, but is frequently a result of inheritance, or, if acquired, it is through years of over-indulgence in eating or drinking. In consequence of this, it will be eradicated but slowly or controlled with difficulty. From time to time exacerbations in the progress of the general constitutional disorder will lead to an exaltation in the local manifestations. At such times the rapid elimination of the uric-acid compounds must be the primary consideration. For this purpose agents which act as solvents of uric acid must be employed; those which the general medical practitioner has found of greatest service are the citrate of lithium and carbonate of potassium. The persistent use of these alkalies it is contended will gradually oxidize the uric acid to the stage of urea or an easily diffusible urate, after which it is easily eliminated by the kidneys. Colchicum has also enjoyed much popularity for many years as an eliminating agent acting on both kidneys and bowels.

The physician maintains that regular and systematic treatment must be established if the constitutional uric-acid diathesis is to be overcome. Now, if it be true, as I have intimated and am inclined to believe, that it is the presence of this uric-acid diathesis which predisposes to, and in many cases gives origin to, the local hæmatogenic calcic pericementitis, the sooner we as practising dentists recognize this fact and modify our treatment to the existing conditions, the more efficiently will we serve our patients.

In presenting this paper with the statements embraced therein, which I have endeavored to demonstrate, I hope it will open a broader field for investigation, which will, in the near future or eventually, lead to a more intelligent and united judgment upon the etiology of pyorrhœa alveolaris, which certainly at the present time is so unsatisfactory.

I cannot close this short address without again expressing my great obligation to my friend and colleague Professor Albert P. Brubaker, M.D., D.D.S., who has not only carefully repeated in my presence the analysis and experiments of Professor Congdon, with the addition of a number of others, but has most cheerfully furnished me with the pathology and therapeutics of uric-acid treatment, making that part of the paper thoroughly consistent with the recognized theories of to-day.

